# Identification of Early Biomarkers during Acetaminophen-Induced Hepatotoxicity by Fourier Transform Infrared Microspectroscopy

**DOI:** 10.1371/journal.pone.0045521

**Published:** 2012-09-19

**Authors:** Rekha Gautam, Bhagawat Chandrasekar, Mukta Deobagkar-Lele, Srabanti Rakshit, Vinay Kumar B. N., Siva Umapathy, Dipankar Nandi

**Affiliations:** 1 Department of Inorganic and Physical Chemistry, Indian Institute of Science, Bangalore, India; 2 Department of Biochemistry, Indian Institute of Science, Bangalore, India; National Institutes of Health, United States of America

## Abstract

Acetaminophen is a widely prescribed drug used to relieve pain and fever; however, it is a leading cause of drug-induced liver injury and a burden on public healthcare. In this study, hepatotoxicity in mice post oral dosing of acetaminophen was investigated using liver and sera samples with Fourier Transform Infrared microspectroscopy. The infrared spectra of acetaminophen treated livers in BALB/c mice show decrease in glycogen, increase in amounts of cholesteryl esters and DNA respectively. Rescue experiments using L-methionine demonstrate that depletion in glycogen and increase in DNA are abrogated with pre-treatment, but not post-treatment, with L-methionine. This indicates that changes in glycogen and DNA are more sensitive to the rapid depletion of glutathione. Importantly, analysis of sera identified lowering of glycogen and increase in DNA and chlolesteryl esters earlier than increase in alanine aminotransferase, which is routinely used to diagnose liver damage. In addition, these changes are also observed in C57BL/6 and *Nos2* ^−/−^ mice. There is no difference in the kinetics of expression of these three molecules in both strains of mice, the extent of damage is similar and corroborated with ALT and histological analysis. Quantification of cytokines in sera showed increase upon APAP treatment. Although the levels of Tnfα and Ifnγ in sera are not significantly affected, *Nos2* ^−/−^ mice display lower Il6 but higher Il10 levels during this acute model of hepatotoxicity. Overall, this study reinforces the growing potential of Fourier Transform Infrared microspectroscopy as a fast, highly sensitive and label-free technique for non-invasive diagnosis of liver damage. The combination of Fourier Transform Infrared microspectroscopy and cytokine analysis is a powerful tool to identify multiple biomarkers, understand differential host responses and evaluate therapeutic regimens during liver damage and, possibly, other diseases.

## Introduction

Drug-induced hepatotoxicity has been attributed to be the cause for a major percentage of patient morbidity and mortality. It is well established that the liver plays an important role in drug metabolism and is, thus, highly susceptible to drug toxicity. Acetaminophen (APAP) is an analgesic and antipyretic drug which is extensively used for therapeutic purposes. The probability of developing liver injury due to consumption of APAP as prescribed is low; however, APAP consumption with alcohol, during fasting and malnutrition for prolonged periods may trigger hepatotoxicity. Also, accidental and intentional over dosing of APAP is a cause of major health concern as it is the main source of acute liver failure in the Western world. Suspected APAP hepatotoxicity can be effectively treated with N-acetylcysteine, yet an estimated 500 patients die each year in the USA [1], [2]. Hence, there is a need to better understand, diagnose and effectively treat cases of APAP-induced hepatotoxicity.

APAP is readily absorbed by the gastrointestinal tract and metabolized by three main pathways: glucuronidation, sulfation and N-hydroxylation and rearrangement. Most of the final metabolic products of APAP are non-toxic and excreted by the urine. However, a minor metabolite known as N-acetyl-p-benzoquinone imine (NAPQI), which is produced due to the action of the liver cytochrome P450 system, is harmful. Several human isoforms of the cytochrome P450 enzymes have been implicated in the bioactivation of APAP and in the generation of intermediates during APAP metabolism [3]. NAPQI conjugates with glutathione (GSH) and is then further metabolized to form cysteine and mercapturic acid conjugates. However, overdose of APAP leads to the saturation of the conjugation pathways and the depletion of GSH induces oxidative stress. Consequently, NAPQI forms covalent bonds with several protein and non-protein thiols leading to cell death [4], [5]. Several biochemical and cellular changes occur during APAP-induced liver damage: increase in free radicals, c-Jun N-terminal Kinase activation, greater mitochondrial dysfunction, nuclear DNA fragmentation, etc. Due to necrosis, endogenous adjuvants are released which trigger the release of cytokines and chemokines [5]. Despite several biochemical and clinical studies, APAP-induced hepatotoxicity has not been completely understood. Serum alanine aminotransferase (ALT) and/or aspartate aminotransferase activity are the most frequently used biomarkers to detect hepatocellular injury in clinical practice. However, there is a crucial need for newer techniques which can provide molecular-level information on functional groups, bonding types, and conformations to help detect and, possibly, treat liver injury at early stages.

A variety of imaging modalities are currently used in medicine such as Magnetic Resonance Imaging, Positron Electron Tomography, Computed Tomography, optical bioluminescence, fluorescence etc [6]. Some of them use X-rays or γ-rays which are destructive to biological samples apart from the need for sample to be tagged using specific dyes or contrast agents that can potentially harm the integrity of the sample under study. Also, a technique like Nuclear Magnetic Resonance needs sample to be homogenized and metabolites isolated in a specific manner in order to obtain comprehensible results. The other main limitations encountered by several imaging techniques are poor sensitivity and resolution [7]–[9]. Therefore, it is not easy to find a single technique which meets the need for high sensitivity and high spatial resolution for specific applications. Different analytical techniques have been used to study APAP-induced toxicity, including Nuclear Magnetic Resonance spectroscopy [10], Mass Spectrometry [11] and Magnetic Resonance Imaging [12].

Recent advances in imaging applications with different degrees of sensitivity and resolution are used in medicine and label free imaging is an emerging technology [8], [13]. Fourier Transform Infrared (FTIR) spectroscopy is one such label free method, in which infrared radiations interact with matter and are selectively absorbed by it according to their chemical composition, thus creating a molecular fingerprint [14]. FTIR microspectroscopy, a combination of FTIR spectroscopy and microscopy, is a proven cutting edge technique applied in the fields of histology, cytology and clinical chemistry to understand biological processes at the molecular level [15], [16]. In addition, FTIR imaging technique has been used for early diagnosis and progression of bacterial and fungal infections and for understanding the underlying chemical and morphological changes in tissue samples such as breast and bone nodal tissues [17]–[19].

During APAP-induced hepatotoxicity, pro-inflammatory cytokines, e.g. Ifnγ, are well known to up-regulate nitric oxide synthase (Nos) 2, resulting in increase in nitric oxide, a key signalling mediator. Increase in nitro-tyrosine adducts in the hepatic centro-lobular cells are observed during APAP-induced liver damage. Also, regulatory circuits control the production of some cytokines via Nos2. However, reports on the functional roles of Nos2 during APAP-induced liver damage are inconsistent [20]–[24]. Studies with specific inhibitors to Nos showed the role of nitric oxide in APAP induced hepatotoxicity [20], [21]. Interestingly, intra-peritoneal injection of APAP in *Nos2* ^−/−^ mice showed reduced [22] or similar [23], [24] susceptibility compared to wild type controls. Also, no difference in liver damage was found in mice lacking Nos2 in a cadmium model of liver toxicity [25]. Hence it was important to address the role of Nos2 in our model of APAP-induced hepatotoxicity with respect to two aspects: functional consequences and regulation of biomarkers. In the present study, some aspects of APAP-induced hepatotoxicity were investigated using FTIR microspectroscopy. Initial experiments were performed using BALB/c mice and the role of *Nos2* during APAP-induced hepatotoxicity was addressed using C57BL/6 and *Nos2* ^−/−^ mice. This study focuses on finding novel markers using FTIR imaging which, coupled with cytokine responses, will be helpful for rapid diagnosis and better understanding of host responses during liver damage.

## Results

### FTIR Spectra Reveal Changes in Liver upon APAP Treatment

**Figure 1:**
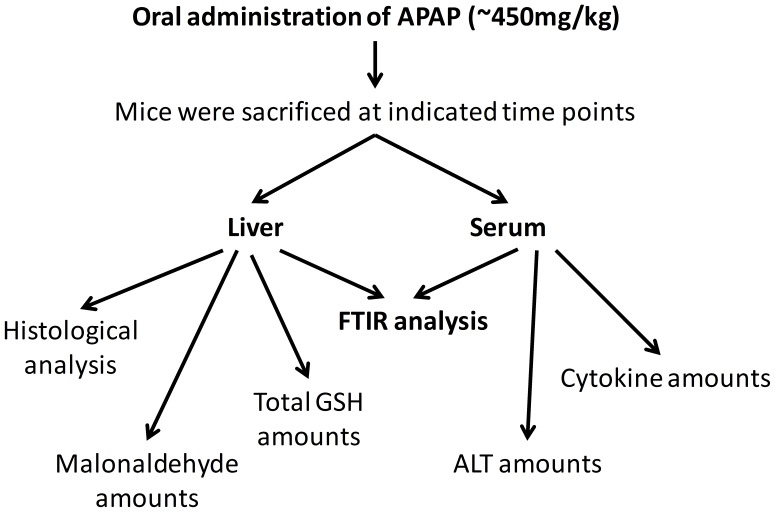
A schematic representation of the experimental strategy and analysis performed in this study.

To obtain novel insights in the APAP induced liver injury process, FTIR spectra were recorded from control and APAP treated BALB/c mice (6 h) livers. A schematic model representing the experimental strategies and analysis is shown (Figure 1). The FTIR spectra (950–3800 cm**^−^** ^1^) were complex (Figure S1A **)** and represented numerous bands arising from the contribution of different functional groups of macromolecules [26], [27]. The tentative assignments of principle IR bands are shown in Table 1.

**Table 1:** Band assignments for the IR spectra [26], [27].

Wavenumber (cm^–1^)	Spectral assignments
966	C-O stretch deoxyribose and C-N^+^-C stretch (mainly from DNA)
996	C-O stretch ribose (mainly from RNA)
1030	C–O stretch (mainly from glycogen)
1080	PO_2_^–^ symmetric stretch in glycogen and nucleic acids
1152	CO–O–C asymmetric stretch (mainly from glycogen)
1171	CO-O-C asymmetric stretch from ester bonds in cholesteryl esters
1238	PO_2_^–^ asymmetric stretch (mainly nucleic acids)
1397	COO^-^ symmetric stretch in fatty acids and amino acids
1451	CH_2_ bending (mainly from lipids)
1542	N–H bending and C–N stretch in proteins (Amide II)
1648	C═O stretch in proteins (Amide I)
1741	C═O stretch in triglycerides and cholesterol esters
2856	CH_2_ symmetric stretch (mainly from lipids)
2875	CH_2_ symmetric stretch (mainly from lipids)
2926	CH_2_ asymmetric stretch (mainly from lipids)
2956	CH_3_ asymmetric stretch (mainly from lipids)
3012	= C-H stretch (mainly from unsaturated lipids)

**Figure 2:**
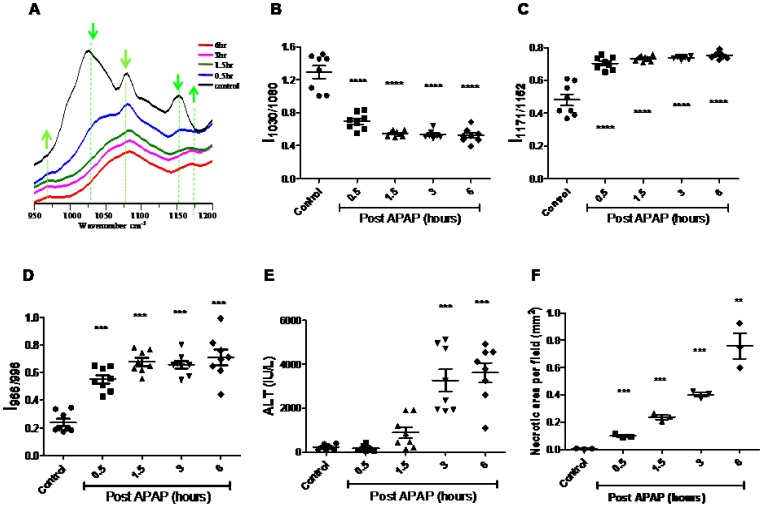
FTIR analysis of APAP induced liver damage in BALB/c mice. FTIR spectra (950 cm**^−^** ^1^ to 1200 cm**^−^** ^1^) of control and APAP treated mice livers at indicated time points. Arrows indicate the wave numbers where differences were observed (A). Kinetic changes in the liver glycogen (B), Cholesteryl ester (C), DNA (D) and ALT (E) amounts in APAP treated mice. The extent of necrosis in control and APAP treated mice livers at indicated time points was quantified after Hematoxylin and eosin staining (F). All the data are shown as mean ± S.E. with n = 3 mice or more.

FTIR spectra indicated changes in the region (950–1200 cm**^−^** ^1^) (Figure 2A **)**. Control livers exhibited a strong absorption at 1030 cm**^−^** ^1^ (C–O stretch), 1080 cm**^−^** ^1^ (PO_2_^–^ symmetric stretch) and 1152 cm**^−^** ^1^ (CO–O–C asymmetric stretch), arising mostly due to glycogen. However, these specific absorption bands for glycogen greatly decreased in APAP treated livers. Also, the ratio of the band intensities at 966 cm**^−^** ^1^ (C-O stretch, deoxyribose, C-N^+^-C stretch) to 996 cm**^−^** ^1^ (C-O stretch, ribose) increased in APAP treated mice livers in comparison to their controls. The band at 1171 cm**^−^** ^1^, corresponding to CO-O-C asymmetric stretching vibration of ester bonds in cholesteryl esters (phospholipids and cholesterol), also increased in APAP treated mice liver in comparison to their controls.

### Bands Corresponding to Glycogen, Cholesteryl Esters and DNA are Kinetically Regulated upon APAP Treatment

**Figure 3:**
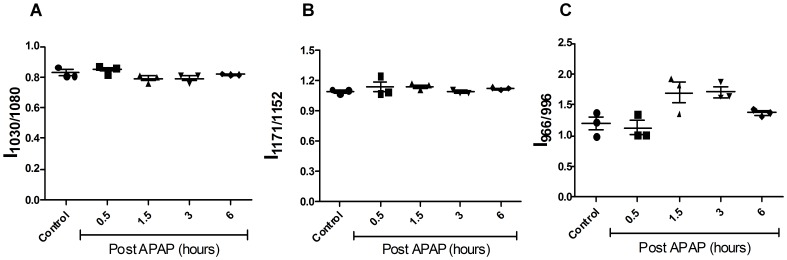
Kinetic analysis of spleen post oral dosing with APAP in mice using FTIR. Changes in glycogen (1030 cm**^−^** ^1^/1080 cm**^−^** ^1^; A), cholesteryl ester (1171 cm**^−^** ^1^/1152 cm**^−^** ^1^; B) and DNA (966 cm**^−^** ^1^/996 cm**^−^** ^1^; C) in sera from APAP treated BALB/c mice. All the data are represented as mean ± S.E. with n = 3 mice.

To understand the regulation of above mentioned markers upon APAP treatment, kinetic experiments were performed. Glycogen (IR bands at 1030 cm**^−^** ^1^, 1080 cm**^−^** ^1^ and 1152 cm**^−^** ^1^) decreased in a time dependent manner upon APAP treatment in liver (Figure 2B and Figure S1C). This decrease was rapid, significant and occurred as early as 0.5 h and did not recover until 6 h post APAP treatment. Esters of cholesterol and phospholipids (IR bands 1171 cm**^−^** ^1^/1152 cm**^−^** ^1^) increased rapidly by 0.5 h and were sustained up to 6 h post APAP treatment (Figure 2C). Also, DNA/RNA ratio (IR band ratio 966 cm**^−^** ^1^/996 cm**^−^** ^1^) increased significantly by 0.5 h, peaking at about 1.5 h and thereafter was sustained up to 6 h post APAP treatment (Figure 2D). It is important to highlight here that the FTIR spectral changes observed (950–1200 cm**^−^** ^1^) between control and APAP treated mice were specific to livers and not to other organs, e.g. spleen (Figure 3).

**Figure 4:**
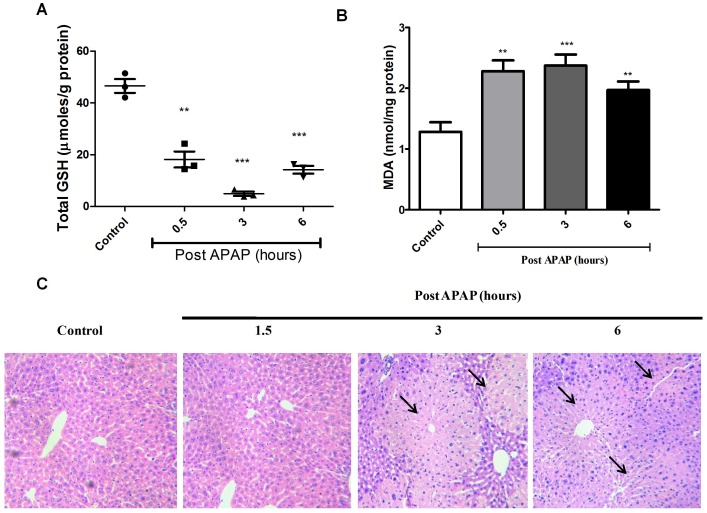
Biochemical and histological analysis of APAP induced liver damage in BALB/c mice. Total GSH amounts (A), malonaldehyde amounts (B) and hematoxylin and eosin staining (C) of livers upon APAP treatment in BALB/c mice. Arrows indicate necrotic lesions (C). Each panel represents data from experiments with n = 3 mice.

The changes observed using FTIR correlated well with biochemical changes that occur quickly with APAP treatment in liver, i.e. significant drop in total GSH (Figure 4A) and rise in malonaldehyde amounts (Figure 4B). On the other hand, liver damage after APAP dosing, as assessed by the increase in amounts of ALT in sera (Figure 2E) and histological examination of liver sections (Figure 2F and Figure 4C), occurred at later time points. Both the biological assays indicated a substantial rise in liver injury only from 3 h post APAP treatment that peaked with maximum damage by 6 h.

### FTIR Analysis of Amelioration of APAP Hepatotoxicity by L-methionine (L-met)

**Figure 5:**
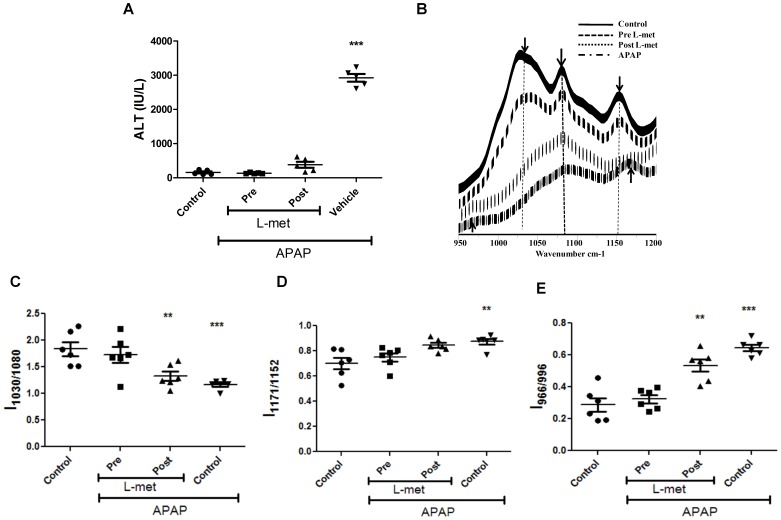
Studies with pre and post L-methionine treatment in APAP treated BALB/c mice. Changes in ALT amounts in sera upon pre and post L-methionine treatment in APAP treated mice (A). FTIR spectra (950 cm**^−^** ^1^ to 1200 cm**^−^** ^1^) of pre- and post- L-methionine treatment in APAP treated mice livers (B). Changes in glycogen (C), cholesteryl ester (D) and DNA (E) amounts upon pre and post L-methionine treatment of APAP treated mice livers. All data are represented as mean ± S.E. with n = 5 or more mice.

To delineate the sequence of molecular changes detected by FTIR, experiments were performed in APAP treated mice with an antidote, e.g. L-met. Both pre (−0.5 h) as well as post (+0.5 h) L-met treatment of APAP treated mice rescued hepatotoxicity as revealed by ALT amounts (Figure 5A). FTIR spectra analysis of livers from these experiments revealed interesting differences between pre and post L-met treated groups (Figure 5B). L-met pre treatment, but not post, restored glycogen (Figure 5C and Figure S2) and DNA (Figure 5E) to levels almost equivalent to control mice. However, in case of cholesteryl esters, both pre and post L-met treatment showed significant rescue (Figure 5D). Most likely, the drop in glycogen levels and increase in DNA amounts are molecular changes that are sensitive to the early rise in oxidative stress.

### FTIR does not Differentiate any Molecular Changes in APAP Treated C57BL/6 and Nos2^−/−^ Mice

**Figure 6:**
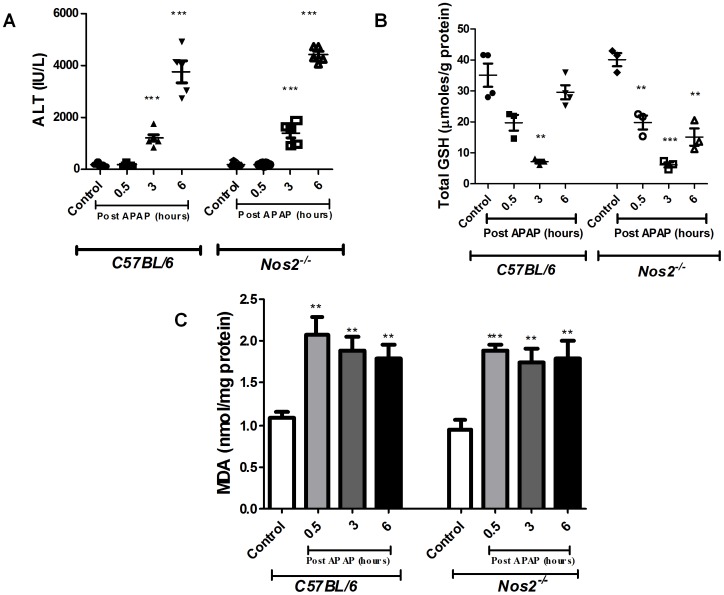
Changes in sera ALT (A), liver GSH (B) and liver malonaldehyde amounts (C) from APAP treated C57BL/6 and *Nos2^−/−^* mice. All the data are represented as mean ± S.E. with n = 3 or more mice.

**Figure 7:**
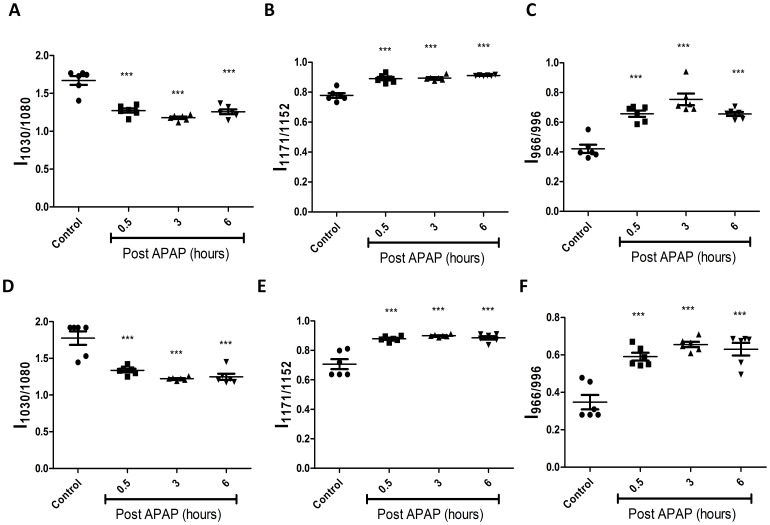
Comparative kinetic analysis of livers from APAP treated C57BL/6 and *Nos2^−/−^* mice. Changes in glycogen (A & D), cholesteryl ester (B & E) and DNA (C & F) from APAP treated C57BL/6 (top panel) and *Nos2^−/−^* (bottom panel) mice livers are shown. All the data are represented as mean ± S.E. with n = 6 mice.

Next, experiments with another mouse strain, C57BL/6 were performed. Since the role of Nos2 during APAP induced hepatotoxicity has been controversial [22]–[24], a comparative kinetic analysis of C57BL/6 and *Nos2^−/−^* mice treated with APAP was performed. Importantly, no difference in the pattern of serum ALT, liver malonaldehyde and GSH amounts (Figure 6) was observed. Also, histological examination of liver sections (Figure S3) indicated that damage was similar in C57BL/6 and *Nos2^−/−^* mice treated with APAP. FTIR spectral analysis (Figure 7) revealed that the pattern of decrease in glycogen levels, increase in cholesteryl ester levels and increase in DNA levels was similar in C57BL/6 and *Nos2^−/−^* mice treated with APAP.

### FTIR Detects Changes in Sera of Mice Dosed with APAP

**Figure 8:**
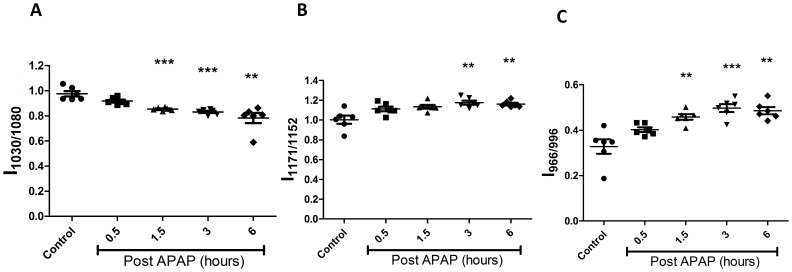
FTIR Analysis of sera from APAP treated BALB/c mice. Changes in the glycogen (1030 cm**^−^** ^1^/1080 cm**^−^** ^1^; A), Cholesteryl ester (1171 cm**^−^** ^1^/1152 cm**^−^** ^1^; B) and DNA (966 cm**^−^** ^1^/996 cm**^−^** ^1^; C) amounts in APAP treated and untreated mice sera. All the data are shown as mean ± S.E. with n = 5 mice or more.

**Figure 9:**
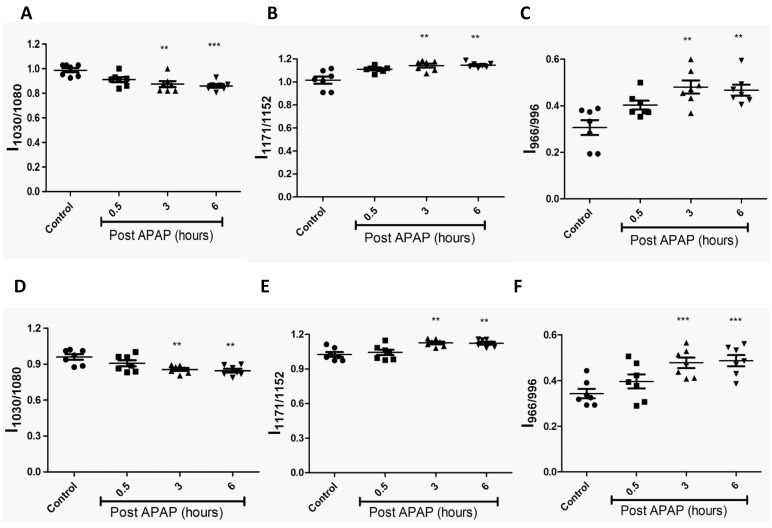
FTIR Analysis of sera from APAP treated C57BL/6 and *Nos2* ^−/−^ mice. Changes in the glycogen (A, D), Cholesteryl ester (B, E) and DNA (C, F) amounts from C57BL/6 (top panel) and *Nos2^−/−^* (bottom panel) mice with time in APAP treated and untreated mice sera. All the data are shown as mean ± S.E. with n = 7 mice.

For ease in detection of liver damage, we used sera of mice treated with APAP for FTIR analysis. Interestingly, similar patterns as observed with liver sections, i.e. decrease in glycogen levels, increase in cholesteryl esters and DNA levels (Figure 8) were found. These data suggest that the pattern of molecular changes detected by FTIR at the site of catabolism of APAP, i.e. liver, could also be detected in sera, although the kinetics was delayed (1.5 h). In addition, these changes were similar in sera of both C57BL/6 and *Nos2^−/−^* mice treated with APAP (Figure 9).

### Modulation of Cytokines during APAP Induced Hepatotoxicity

**Figure 10:**
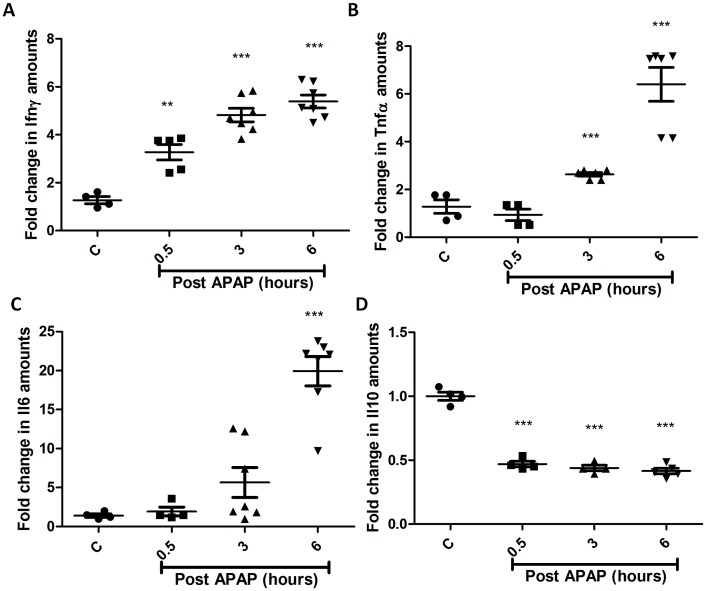
Cytokine analysis in sera of BALB/c mice during APAP-induced liver damage. Fold changes in the levels of Ifnγ (A), Tnfα (B), Il6 (C) and Il10 (D) with time in APAP treated with respect to untreated mice sera. All the data are shown as mean ± S.E. with n = 5 mice or more.

**Figure 11:**
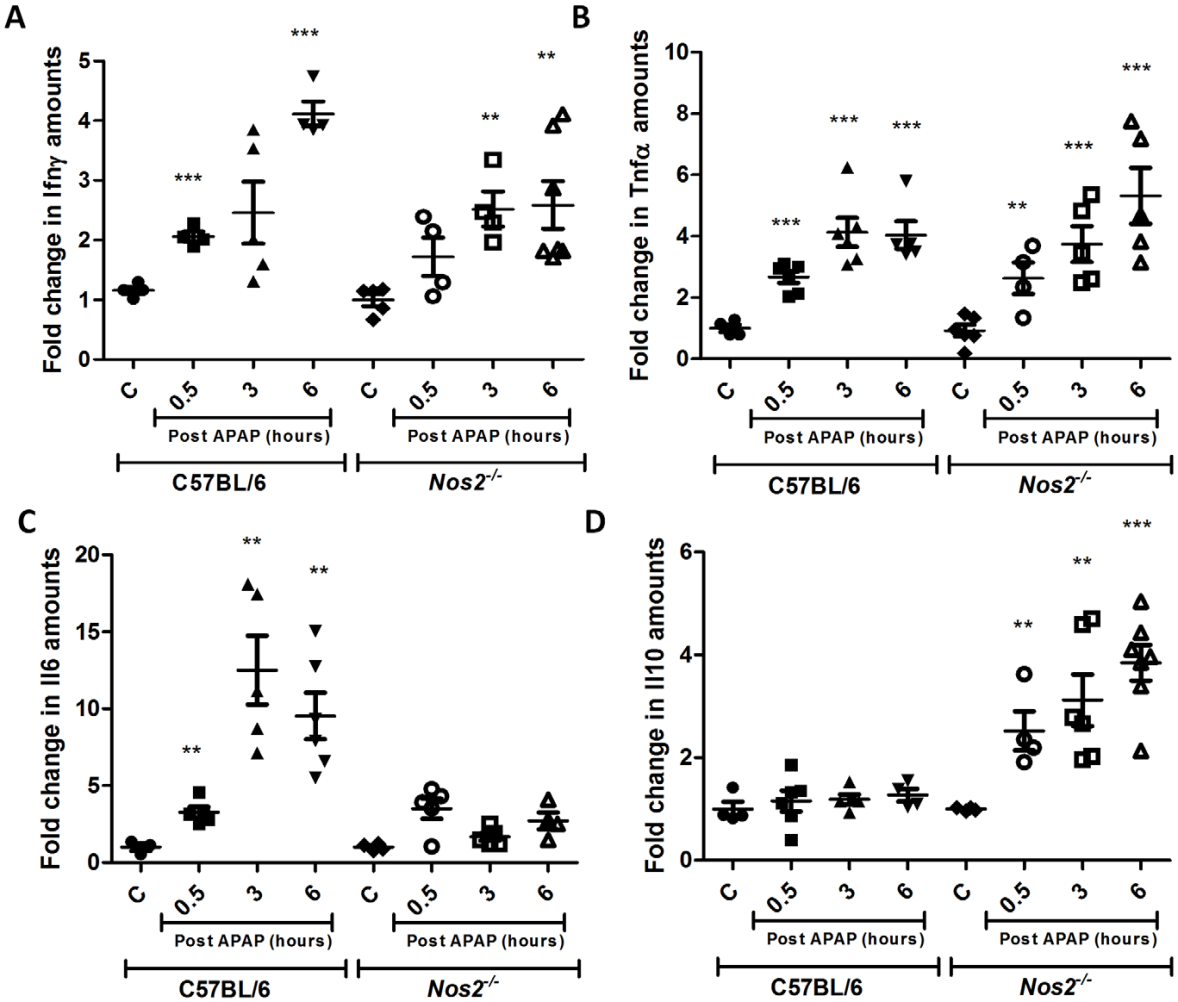
Nos2 modulates the amounts of Il6 and Il10 during APAP induced liver damage. Fold changes in the levels of Ifnγ (A), Tnfα (B), Il6 (C), and Il10 (D) in sera from APAP treated and controls of C57BL/6 and *Nos2^−/−^* mice. All the data are represented as means ± S.E. with n = 5 or more mice.

Analysis of the immune response in terms of serological analysis of cytokines involved in APAP hepatotoxicity was also performed. Serum levels of Ifnγ, Tnfα and Il6 increased but Il10 levels decreased with time post APAP treatment in BALB/c mice (Figure 10). Cytokine analysis in the sera of C57BL/6 and *Nos2^−/−^* mice treated with APAP revealed some differences. The pattern of changes in Ifnγ (Figure 11A) and Tnfα (Figure 11B) in sera were not different in C57BL/6 and *Nos2^−/−^* mice treated with APAP. However, Il6 levels (Figure 11C) increased with time upon APAP treatment in C57BL/6 mice but did not increase as much in *Nos2^−/−^* mice at later time points post APAP dosing. Interestingly, in *Nos2^−/−^* mice treated with APAP, serum Il10 levels (Figure 11D) increased with time unlike in C57BL/6 mice.

## Discussion

There are three aspects to this study involving oral dosing of mice, the physiological route of entry, with APAP: First is the feasibility of using FTIR spectroscopy to diagnose APAP induced liver toxicity with high sensitivity using liver samples or sera. Second is the decrease in glycogen and increase in DNA as molecular changes that are highly sensitive to lowering of GSH amounts which probably leads to oxidative stress. In the third part, cytokine analysis of sera revealed the role of Nos2 in modulating some cytokines, i.e. Il6 and Il10.

The FTIR spectral data analysis detected the changes in injured mice liver as early as 0.5 h (Figure 2 and Figure S1) and these changes were specific to the liver and not spleen (Figure 3). There was an early and significant drop in liver glycogen amounts that remained low over time. While the drop in glycogen was specific to liver and sera but not spleen, upon APAP-induced hepatotoxicity, lower glycogen amounts is also observed in CCl_4_ induced hepatotoxicity and hepatectomy [28], [29]. On the other hand, in cases of HCV infection and liver cirrhosis, glycogen amounts in liver increases [30], [31]. In fact, glycogen indeed corresponds to the decrease in the ratio of wave numbers 1030 and 1080 using FTIR microspectroscopy was confirmed using purified glycogen (Figure S4). Therefore, the drop in glycogen amounts during APAP-induced hepatotoxicity should be included along with other markers, e.g. increase in DNA, cholesteryl esters, ALT etc to diagnose disease progression. The sustained depletion in glycogen may be due to multiple reasons: First, mitochondrial respiration is known to be impaired upon APAP induced hepatotoxicity [32], which may lower glycogen amounts due to enhanced glycolysis. Second, studies using inhibitors and genetic knockout mice which results in low glutathione amounts also lowers the cellular glycogen pool. For example, low glutathione amounts decreases the activity of enzymes involved in glycogen metabolism (e.g. glycogen synthase), thus lowering glycogen amounts [33]. Also, in hepatocytes, upon treatment with GSH deficiency inducing agents like menadione and BSO causes decrease in cellular pools of glycogen [34]. Finally, astrocytes from glutamate cysteine ligase modulatory subunit knockout mice contain less endogenous amounts of GSH, also display lower glycogen amounts compared to the wild type astrocytes [35]. Our studies with pre and post L-met treatment of mice dosed with APAP also showed that glycogen decrease was dependent on early GSH depletion and was abolished upon pre–treatment of mice with L-met (Figure 5). This aspect is important as both L-met and N-acetylcysteine are well known to feed into GSH synthesis pathway [36].

The presence of a band at 1171 cm**^−^** ^1^ due to CO-O-C asymmetric stretching vibration of ester bonds in cholesteryl esters indicates disturbance in lipid metabolism. Cholesteryl esters are formed due to the linkage of cholesterol with fatty acid acyl coenzyme A. Increase in cholesteryl esters is associated with obesity, degeneration of the hippocampus and cancerous tissue due to increased proliferation [37], [38]. Interestingly, Toyran *et al.* observed that intensity of the band at 1171 cm^−1^ decreases whereas the band at 1151 cm^−1^ increases in the diabetic group with respect to the control [26]. Most likely, the increase in the intensity of the band at 1151 cm^−1^ in the diabetic group is due to increase in glycogen. It is known that high amounts of plasma non-esterified fatty acids cause glycogen accumulation due to alterations in carbohydrate and lipid metabolism during diabetes. Further studies are required to understand the physiological implications of the increase in cholesteryl esters during liver damage.

The increase in 966/996 cm**^−^** ^1^ ratio i.e. the increase in DNA has at least three potential explanations: (i) proliferation of cells, (ii) inflammatory cell infiltration and (iii) necrosis and apoptosis at the site of liver injury. The probability that the rise in DNA was due to increased proliferation of cells was low as, only 10 out of 20,000 liver cells undergo division since the majority are mitotically inactive [39]. Also, liver regeneration occurs much later after injury, ∼72 h after initiation of damage [39], [40]. The infiltration of inflammatory cells during liver damage was negligible in this model (Figure 4C and Figure S3). Most likely, the increase in detection of DNA was due to cell death as a possible consequence of the translocation of Bax into the mitochondria [41].

The role of preventive or therapeutic treatment for liver damage was addressed by injecting L-met before or after APAP dosing. Liver damage as assessed by studying ALT amounts in the sera indicated complete recovery under both conditions. However, FTIR analysis clearly revealed differences and, with post L-met treatment, reduction in glycogen and increase in DNA were clearly observed (Figure 5). Most likely, these two changes were more sensitive to early increase in oxidative stress. This study has clearly defined the molecular changes that are differentially responsive to early or late changes in the GSH amounts in livers undergoing injury.

The cellular and biochemical processes associated with APAP-induced liver damage are well studied [1], [4], [5]. Both glycogen and GSH are known to be greatly reduced following APAP administration [42]. Also, DNA is released in the sera following liver damage and has been used as a marker [43]. Spectral changes observed in case of liver tissue were also observed with sera although the kinetics were delayed, e.g. 1.5 h compared to 0.5 h (Figure 2 and Figure 8). However, rise in ALT amounts in the sera was observed much later, e.g. 3 h after APAP dosing (Figure 2E and Figure 6). Importantly, in this model of acute hepatotoxicity, FTIR spectroscopy was able to consistently identify different molecules, e.g. glycogen, lipids, nucleic acids etc., while using the same sample. Notably, FTIR microspectroscopy analysis did not identify other markers that have been reported to be associated with APAP induced hepatotoxicity, e.g. increase in collagen (1395 cm**^−^** ^1^), consistently across samples [44]. This aspect of using multiple markers to monitor disease progression may be more reliable than using a single marker. In fact the addition of new biomarkers to existing ones to predict disease susceptibility of patients is of growing interest. For example, in cardiovascular diseases, the use of a single biomarker is not definitive in predicting the progression or susceptibility to disease [45]. In addition, this study reinforces the ability of FTIR to analyze heterogeneous tissue samples to closely study the complex injury process. FTIR studies related to liver toxicity due to various chemicals like Cd^2+^ and CCl_4_ have been reported wherein liver tissue samples were investigated in powdered or homogenized extracts [46], [47]. However, this is the first report of the use of FTIR to study APAP-induced liver damage using cryosectioned tissue, thereby avoiding any possible alterations of the sample. FTIR is very sensitive and detected very early biochemical changes in both liver as well as sera compared with existing biochemical assays. This study highlights the potential of using FTIR as a single non-invasive alternative to the more established individual biochemical assays to monitor disease progression. Furthermore, FTIR microspectroscopy can be used to identify novel biochemical markers in APAP induced hepatotoxicity and other diseases that can be eventually validated using standard biochemical assays or methods. It needs to be highlighted that the changes in biomarkers observed in this study are only in mice and cannot be directly extrapolated to human patients with APAP induced hepatotoxicity. Further studies will be required to identify and validate these biomarkers using FTIR microspectroscopy in other liver injury models and, more importantly, in patients with liver injury and disease.

As there are conflicting reports on the role of Nos2 in modulating APAP-induced liver damage [22]–[24], experiments were designed to address this issue. Unlike a previous report that showed that ALT amounts are lower but lipid peroxidation is increased in *Nos2* ^−/−^ mice upon APAP dosing [23], no differences were observed with respect to FTIR spectroscopy, GSH, malonaldehyde, ALT amounts and histology between C57BL/6 and *Nos2* ^−/−^ mice (Figure 6, Figure 7, Figure 9 and Figure S3); however, differences were observed with some cytokines (Figure 11). Differential cytokine modulation has been shown in the APAP model of liver toxicity. In fact, the higher expression of Il6 in mice has been associated with reduced liver damage [48]. Il6 is known as a hepatocyte growth factor and is important for liver regeneration [39]. In fact, Il6 increases faster in C57BL/6 mice whereas Il10 is reduced in BALB/c mice (Figure 10 and Figure 11). It appears that Nos2 increased Il6 but decreased Il10 levels during APAP-induced hepatotoxicity (Figure 11). Interestingly, nitric oxide has been shown to inhibit the production of Il6 in rat Kupffer cells [49]. In an endotoxin model of lung inflammation, Nos2 has been shown to be important in decreasing Il12 and increasing Il10 amounts in the lung [50]. Also, the presence of Nos2 has been shown to reduce Tnfα and increase Il10 during APAP-induced liver damage [22]. Several reports have shown that Il10 reduces Nos2 induction, which in turn reduces inflammatory responses [51], [52]. Nos2 also regulates Il10 and this aspect is interesting as mice lacking Il10 are shown to be more susceptible to APAP-induced liver damage most likely due to increased production of Nos2 and pro-inflammatory cytokines that increase immunopathology [24].

Overall, this study demonstrates the efficacy of using sera combined with FTIR and cytokine analysis as a non-invasive, rapid and sensitive assay to diagnose liver damage. The cytokine studies are important as they reveal the potential role of differential genetic predispositions in modulating immune responses during liver diseases. Overall, the combination of FTIR and cytokine analysis will open new avenues for rapid diagnosis and a more comprehensive understanding of liver injury and, perhaps, other diseases.

## Materials and Methods

### Animal Ethics Statement

All mice experiments were performed in accordance with the Ministry of Environment and Forests Act regarding the breeding of and experiments on animals (control and supervision) rules, 1998. Mice were bred and maintained by the Central Animal Facility, IISc (Registration number: 48/1999/CPCSEA, dated 1/3/1999), which is accredited to the Ministry of Environment and Forests, Government of India. The details of guidelines issued by CPCSEA can be accessed on the web using the link: http://envfor.nic.in/divisions/awd/cpcsea_laboratory.pdf. All mice experiments were conducted as per the guidelines issued by the Institutional Animal Ethics Committee, IISc (permit no. CAF/Ethics/155/2009). Approval by the Committee for Purpose and Control and Supervision on experiments on animals (CPCSEA) was sought and approved for the protocols used for experimentation.

### Mice

Six to eight week old BALB/c, C57BL/6 and *Nos2^−/−^* mice (18–25 g) were obtained from the Central Animal Facility of the Institute. All mice were provided humane care and experiments were performed as per the guidelines laid out by the institutional animal ethics committee. Mice were starved overnight, orally fed with ∼450 mg/kg APAP in autoclaved milliQ water and sacrificed at indicated time points, as described previously [53]. Mice dosed with autoclaved milliQ water were used as controls. L-met was administered intraperitoneally 0.5 hours (h) before (pre) or 0.5 h after (post) APAP dosing.

### Chemicals

APAP and L-met were obtained from Sigma Aldrich Ltd. (St. Louis, MO). Purified Glycogen was obtained from Sisco Research Laboratories (Mumbai, India). Liver injury was assessed by measuring serum ALT activity using a kit from Coral Clinical Systems (Goa, India).

### FTIR Measurements

Liver and spleen were dissected and tissue samples were washed with PBS, snap frozen in liquid nitrogen and immediately stored at −80°C until further analysis. The tissues were cryosectioned (4 µm thickness) at −20°C and sections were mounted on IR reflective slides (Varian, Inc., MA). IR images of liver and spleen samples were recorded using a commercial FTIR micro-spectrometer (Varian, FTS 7000) coupled with a focal plane array and liquid N_2_-cooled Mercury Cadmium Telluride (MCT) linear detector. All images were recorded at a resolution of 4 cm**^−^** ^1^ and for each image, 64 scans/interferograms were co-added. Images were recorded from 10 different sites for each sample and 50 spectra were averaged from each image. For FTIR analysis of sera, 2 µl of sera were diluted to 5 µl with water and a drop of the same was added on a slide and air dried at 37°C. In case of sera, MCT linear detector was used for recording IR spectrum. A multipoint linear baseline was subtracted from each spectrum and spectra were normalized to the amide II band (1542 cm**^−^** ^1^) as it remained unchanged across different samples and experiments. Tissue or sera changes are represented as glycogen (1030 cm**^−^** ^1^/1080 cm**^−^** ^1^), cholesteryl ester (1171 cm**^−^** ^1^/1152 cm**^−^** ^1^) and DNA (966 cm**^−^** ^1^/996 cm**^−^** ^1^) amounts. All the experiments were performed at least three times with three or more animals per group. Cumulated results across experiments have been presented in figures as mean ± S.E.

### GSH Estimation

Ellman’s reagent was used to quantify the total GSH amounts in the APAP treated and control mice livers, as described previously [53]. The assay involves the reduction of 5, 5′-dithiobis 2-nitrobenzoic acid to a yellow product by sulfhdryl groups present in GSH. In brief, liver samples were homogenized (in 1 ml) in 5% trichloroacetic acid and homogenates were centrifuged at 10,000 g for 30 min at 4°C. GSH in the supernatant was quantified using the extinction coefficient (13,600 M**^−^** ^1 ^cm**^−^** ^1^) of DTNB and normalized to the protein in each sample.

### Histological Analysis

Liver tissues were dissected from mice and fixed overnight in 10% neutral formalin buffer. Sample blocks were prepared by embedding liver tissues in paraffin wax, and sections were stained with haematoxylin-eosin (H & E). Tissue sections were observed using a light microscope and photographs were taken using a Nikon camera fitted to the microscope. Briefly, necrotic lesions were examined using low-power (250 X) light microscopy and images were obtained using a digital camera. Quantification of hepatic necrosis upon APAP treatment was performed as previously reported [54].

### Malonaldehyde Quantification

The quantification of malonaldehyde amounts in the liver samples was performed as described previously [55]. Briefly, liver tissue samples were obtained at different time points and homogenized in 1.15% KCl buffer. The homogenates were centrifuged and 0.8% TBA, 0.25 N HCl and 10% TCA were added to the supernatants. The mixture was incubated in boiling water bath for 20 min, centrifuged and optical density at 535 nm was measured using a micro plate reader (Versa Max, Molecular Devices). The amount of malonaldehyde was calculated using a 1,1,3,3, tetra methoxy propane as external standard. The malonaldehyde amounts were normalized with the protein content in each sample.

### Cytokine Analysis

Sera obtained from mice in different experiments were used to quantify the amounts of the following individual cytokines using ELISA kits (eBioscience, U.S.A): Ifnγ, Tnfα, Il6 and Il10. The samples were diluted and the amounts detectable were in the linear range of standards for each of the cytokines. The manufacturer’s protocol was followed and the colorimetric reaction was developed using TMB substrate, the amounts were calculated from the absorbance measured at 450 nm using a micro plate reader (Versa Max, Molecular Devices). Amounts were calculated and represented as fold change with respect to corresponding controls without APAP.

### Statistical Tests

Data are expressed as mean ± SE. Statistical analysis was performed using Student’s *t* test using GraphPad Prism (5.0) software. The degree of significance was represented by “asterisks” (*) for p<0.05 = *, p<0.01 = **, p<0.001 = ***, p<0.0001 = ****.

## Supporting Information

Figure S1 **FTIR spectra (950 cm^−1^ to 3800 cm^−1^) of control and APAP treated mice livers.** Arrows indicate regions of observable difference (A). Kinetic FTIR spectra (950 cm**^−^** ^1^ to 1200 cm**^−^** ^1^) of control and APAP treated mice spleen at indicated time points (B). FTIR images of control and APAP treated mice livers; both white light and glycogen distribution are shown in following order: (i) Control, (ii) 0.5 h, (iii) 1.5 h, (iv) 3 h, and (v) 6 h – post APAP treatment (C). Each image is a representative across experiments with n = 3 to 6 mice.
(TIF)

Figure S2 **FTIR images of ∼4 µm liver sections of the following order: (i) Control, (ii) pre L-methionine treatment (−0.5 h), (iii) post L-methionine treatment (+0.5 h), (iv) APAP alone.** All images represent data across many experiments with n = 5 or more mice.
(TIF)

Figure S3 **Hematoxylin and eosin stained liver sections from APAP treated and controls of C57BL/6 and** ***Nos2^−/−^*** **mice.** Arrows indicate necrotic lesions. All images represent data across many experiments with n = 3 or more mice.
(TIF)

Figure S4 **FTIR spectra (950 cm^−1^ to 1175 cm^−1^) of glycogen solution in milliQ water (black), control mice liver (blue) and 6 h post APAP treatment mice liver (red).** Arrows indicate regions of observable difference.
(TIF)

## References

[1] Fontana RJ (2008) Acute liver failure due to drugs. Semin Liver Dis. 28: 175–187. 10.1055/s-2008-1073117 18452117

[2] Larson MA, Polson J, Fontana JR, Davern JT, Lalani E, et al (2005) Acetaminophen-induced acute liver failure: results of a United States multicenter, prospective study. Hepatology 42: 1364–1372. 16317692 10.1002/hep.20948

[3] Dong H, Haining RL, Thummel KE, Rettie AE, Nelson SD (2000) Involvement of human cytochrome P450 2D6 in the bioactivation of acetaminophen. Drug Metab Dispos. 28: 1397–1400. 11095574

[4] Jaeschke H, Bajt ML (2006) Intracellular signalling mechanisms of acetaminophen-induced liver cell death. Toxicol Sci. 89: 31–41. 10.1093/toxsci/kfi336 16177235

[5] Jaeschke H, Williams CD, Ramachandran A, Bajt ML (2012) Acetaminophen hepatotoxicity and repair: the role of sterile inflammation and innate immunity. Liver Int. 32: 8–20. 10.1111/j.1478-3231.2011.02501.x PMC3586825 21745276

[6] Baker M (2010) Whole-animal imaging: The whole picture. Nature 463: 977–980. 20164931 10.1038/463977a

[7] Espina R, Yu L, Wang J, Tong Z, Vashishtha S, et al (2009) Nuclear magnetic resonance spectroscopy as a quantitative tool to determine the concentrations of biologically produced metabolites: implications in metabolites in safety testing. Chem Res Toxicol. 22: 299–310. 10.1021/tx800251p 18980340

[8] Sahu KR, Mordechai S (2005) Fourier Transform Infrared Spectroscopy in Cancer Detection. Future Oncol. 1: 635–647. 10.2217/14796694.1.5.635 16556041

[9] Keren S, Zavaleta C, Cheng Z, de la Zerda A, Gheysens O, et al (2008) Noninvasive molecular imaging of small living subjects using Raman spectroscopy. Proc Natl Acad Sci. USA 105: 5844–5849. 10.1073/pnas.0710575105 PMC2299220 18378895

[10] Schoonen WG, Kloks CP, Ploemen JP, Horbach GJ, Smit MJ, et al (2007) Sensitivity of ^(1)^H NMR analysis of rat urine in relation to toxicometabonomics. Part I: dose-dependent toxic effects of bromobenzene and paracetamol. Toxicol Sci. 98: 271–285. 10.1093/toxsci/kfm076 17420223

[11] Sun J, Schnackenberg LK, Beger RD (2009) Studies of acetaminophen and metabolites in urine and their correlations with toxicity using metabolomics. Drug Metab Lett. 3: 130–136. 10.2174/187231209789352139 19702550

[12] Brown AT, Ou X, James LP, Jambhekar K, Pandey T, et al (2012) Correlation of MRI findings to histology of acetaminophen toxicity in the mouse. Magn Reson Imaging. 30: 283–289. 10.1016/j.mri.2011.09.023 PMC3254831 22055850

[13] Chan JW, Lieu DK (2009) Label-free biochemical characterization of stem cells using vibrational spectroscopy. J Biophoton. 2: 656–668. 10.1002/jbio.200910041 19653219

[14] Griffiths RP, de Haseth, AJ (2007) Fourier Transform Infrared Spectrometry. John Wiley & Sons, Inc., Hoboken, New Jersey, USA.

[15] Naumann D, Helm D, Labischinski H (1991) Microbiological characterizations by FT-IR spectroscopy. Nature 351: 81–82. 1902911 10.1038/351081a0

[16] Fernandez CD, Bhargava R, Hewitt MS, Levin WI (2005) Infrared spectroscopic imaging for histopathologic recognition. Nat Biotechnol. 23: 469–474. 10.1038/nbt1080 15793574

[17] Erukhimovitch V, Pavlov V, Talyshinsky M, Souprun Y, Huleihel M (2005) FTIR microscopy as a method for identification of bacterial and fungal infections. J Pharm Biomed Anal. 37: 1105–1108. 10.1016/j.jpba.2004.08.010 15862692

[18] Kidder LH, Kalasinsky VF, Luke JL, Levin IW, Lewis EN (1997) Visualization of silicone gel in human breast tissue using new infrared imaging spectroscopy. Nat Med. 3: 235–237. 10.1038/nm0297-235 9018246

[19] Aydin HM, Hu B, Suso JS, El Haj A, Yang Y (2011) Study of tissue engineered bone nodules by Fourier transform infrared spectroscopy. Analyst. 136: 775–780. 10.1039/c0an00530d 21152629

[20] Kamanaka Y, Kawabata A, Matsuya H, Taga C, Sekiguchi F, et al (2003) Effect of a potent iNOS inhibitor (ONO-1714) on acetaminophen-induced hepatotoxicity in the rat. Life Sci. 74: 793–802. 10.1016/j.lfs.2003.09.036 14654171

[21] Gardner CR, Heck DE, Yang CS, Thomas PE, Zhang XJ, et al (1998) Role of nitric oxide in acetaminophen-induced hepatotoxicity in the rat. Hepatology 27: 748–754. 9500703 10.1002/hep.510270316

[22] Gardner CR, Laskin JD, Dambach DM, Sacco M, Durham SK, et al (2002) Reduced hepatotoxicity of acetaminophen in mice lacking inducible nitric oxide synthase: potential role of tumor necrosis factor-alpha and interleukin-10. Toxicol Appl Pharmacol. 184: 27–36. 12392966

[23] Michael SL, Mayeux PR, Bucci TJ, Warbritton AR, Irwin LK, et al (2001) Acetaminophen-induced hepatotoxicity in mice lacking inducible nitric oxide synthase activity. Nitric Oxide 5: 432–441. 11587558 10.1006/niox.2001.0385

[24] Bourdi M, Masubuchi Y, Reilly TP, Amouzadeh HR, Martin JL, et al (2002) Protection against acetaminophen-induced liver injury and lethality by interleukin 10: role of inducible nitric oxide synthase. Hepatology 35: 289–298. 11826401 10.1053/jhep.2002.30956

[25] Harstad EB, Klaassen CD (2002) iNOS-null mice are not resistant to cadmium chloride-induced hepatotoxicity. Toxicology 175: 83–90. 12049838 10.1016/s0300-483x(02)00068-9

[26] Toyran N, Lasch P, Naumann D, Turan B, Severcan F (2006) Early alterations in myocardia and vessels of the diabetic rat heart: an FTIR microspectroscopic study. Biochem J. 397: 427–436. 10.1042/BJ20060171 PMC1533317 16719841

[27] Movasaghi Z, Rehman S, Rehman ur I (2008) Fourier Transform Infrared (FTIR) Spectroscopy of Biological Tissues. Applied Spectroscopy Reviews 43: 134–179.

[28] Lockard VG, Mehendale HM, O’Neal RM (1983) Chlordecone-induced potentiation of carbon tetrachloride hepatotoxicity: a light and electron microscopic study. Exp Mol Pathol. 39: 230–245. 10.1016/0014-4800(83)90054-0 6194012

[29] Kasahara H, Ohyanagi H, Saitoh Y (1988) Changes of gluconeogenesis and alanine metabolism following partial hepatectomy in normal and cirrhotic rats. Nihon Geka Gakkai Zasshi. 89: 365–375. 3393128

[30] Chiriboga L, Yee H, Diem M (2000) Infrared Spectroscopy of Human Cells and Tissue. Part VI: A Comparative Study of Histopathology and Infrared Microspectroscopy of Normal, Cirrhotic, and Cancerous Liver Tissue. Appl Spectrosc. 54: 1–8.

[31] Tarasów E, Wiercińska-Drapało A, Jaroszewicz J, Siergiejczyk L, Orzechowska-Bobkiewicz A, et al (2004) Metabolic disturbances in liver 1H MR spectroscopy in HIV and HCV co-infected patients as a potential marker of hepatocyte activation. Acta Radiol. 45: 803–809. 10.1080/02841850410008711 15690608

[32] Katyare SS, Satav JG (1989) Impaired mitochondrial oxidative energy metabolism following paracetamol-induced hepatotoxicity in the rat. Br J Pharmacol. 96: 51–58. 10.1111/j.1476-5381.1989.tb11783.x PMC1854327 2522334

[33] Braun L, Csala M, Poussu A, Garzo T, Mandl J, et al (1996) Glutathione depletion induces glycogenolysis dependent ascorbate synthesis in isolated murine hepatocytes. FEBS Letters 388: 173–176. 8690080 10.1016/0014-5793(96)00548-0

[34] Braun L, Kardon T, Puskás F, Csala M, Bánhegyi G, et al (1997) Regulation of glucuronidation by glutathione redox state through the alteration of UDP-glucose supply originating from glycogen metabolism. Arch Biochem Biophys. 348: 169–173. 10.1006/abbi.1997.0379 9390188

[35] Lavoie S, Allaman I, Petit JM, Do KQ, Magistretti PJ (2011) Altered glycogen metabolism in cultured astrocytes from mice with chronic glutathione deficit; relevance for neuroenergetics in schizophrenia. PLoS One 6: e22875. 21829542 10.1371/journal.pone.0022875 PMC3145770

[36] Lu SC (1999) Regulation of hepatic glutathione synthesis: current concepts and controversies. FASEB J. 13: 1169–1183. 10385608

[37] Kim JH, Ee SM, Jittiwat J, Ong ES, Farooqui AA, et al (2011) Increased expression of acyl-coenzyme A: cholesterol acyltransferase-1 and elevated cholesteryl esters in the hippocampus after excitotoxic injury. Neuroscience 185: 125–134. 21514367 10.1016/j.neuroscience.2011.04.018

[38] Tosia RM, Tugnoli V (2005) Cholesteryl esters in malignancy. Clinica Chimica Acta 359: 27–45. 10.1016/j.cccn.2005.04.003 15939411

[39] James PL, Lamps WL, McCullough S, Hinson AJ (2003) Interleukin 6 and hepatocyte regeneration in acetaminophen toxicity in the mouse. Biochemical and Biophysical Research Communications 309: 857–863. 13679052 10.1016/j.bbrc.2003.08.085

[40] Grypioti DA, Theocharis ES, Demopoulos AC, Papadopoulou-Daifoti Z, Basayiannis CA, et al (2006) Effect of platelet-activating factor (PAF) receptor antagonist (BN52021) on acetaminophen-induced acute liver injury and regeneration in rats. Liver Int. 26: 97–105. 10.1111/j.1478-3231.2005.01186.x 16420515

[41] Bajt ML, Farhood A, Lemasters JJ, Jaeschke H (2008) Mitochondrial bax translocation accelerates DNA fragmentation and cell necrosis in a murine model of acetaminophen hepatotoxicity. J Pharmacol Exp Ther. 324: 8–14. 10.1124/jpet.107.129445 17906064

[42] Hinson JA, Mays JB, Cameron AM (1983) Acetaminophen-induced hepatic glycogen depletion and hyperglycemia in mice. Biochem Pharmacol. 32: 1979–1988. 10.1016/0006-2952(83)90415-x 6870927

[43] Tran TT, Groben P, Pisetsky DS (2008) The release of DNA into the plasma of mice following hepatic cell death by apoptosis and necrosis. Biomarkers 13: 184–200. 18270870 10.1080/13547500701791719

[44] Sener G, Toklu HZ, Sehirli AO, Velioğlu-Oğünç A, Cetinel S, et al (2006) Protective effects of resveratrol against acetaminophen-induced toxicity in mice. Hepatol Res. 35: 62–68. 10.1016/j.hepres.2006.02.005 16595188

[45] Zethelius B, Berglund L, Sundström J, Ingelsson E, Basu S, et al (2008) Use of multiple biomarkers to improve the prediction of death from cardiovascular causes. N Engl J Med. 358: 2107–2116. 10.1056/NEJMoa0707064 18480203

[46] Henczova M, Aranka Kiss Deer KA, Komlosi V, Mink J (2006) Detection of toxic effects of Cd^2+^ on different fish species via liver cytochrome P450-dependent monooxygenase activities and FTIR spectroscopy. Anal Bioanal Chem. 385: 652–659. 10.1007/s00216-006-0429-y 16715285

[47] Melin AM, Perromat A, Deleris G (2000) Pharmacologic Application of Fourier Transform IR Spectroscopy: In Vivo Toxicity of Carbon Tetrachloride on Rat Liver. Biopolymers 57: 160–168. 10805913 10.1002/(SICI)1097-0282(2000)57:3<160::AID-BIP4>3.0.CO;2-1

[48] Masubuchi Y, Sugiyama S, Horie T (2009) Th1/Th2 cytokine balance as a determinant of acetaminophen-induced liver injury. Chem Biol Interact. 179: 273–279. 10.1016/j.cbi.2008.10.028 19014921

[49] Stadler J, Harbrecht BG, Di Silvio M, Curran RD, Jordan ML, et al (1993) Endogenous nitric oxide inhibits the synthesis of cyclooxygenase products and interleukin-6 by rat Kupffer cells. J Leukoc Biol. 53: 165–172. 10.1002/jlb.53.2.165 8445328

[50] Shanley TP, Zhao B, Macariola DR, Denenberg A, Salzman AL, et al (2002) Role of nitric oxide in acute lung inflammation: lessons learned from the inducible nitric oxide synthase knockout mouse. Crit Care Med. 30: 1960–1968. 10.1097/00003246-200209000-00003 12352027

[51] Ameredes BT, Zamora R, Gibson KF, Billiar TR, Dixon-McCarthy B, et al (2001) Increased nitric oxide production by airway cells of sensitized and challenged IL-10 knockout mice. J Leukoc Biol. 70: 730–736. 11698492

[52] Szalay G, Sauter M, Hald J, Weinzierl A, Kandolf R, et al (2006) Sustained nitric oxide synthesis contributes to immunopathology in ongoing myocarditis attributable to interleukin-10 disorders. Am J Pathol. 169: 2085–2093. 10.2353/ajpath.2006.060350 PMC1762471 17148671

[53] Saha B, Nandi D (2009) Farnesyltransferase Inhibitors Reduce Ras Activation and Ameliorate Acetaminophen-Induced Liver Injury in Mice. Hepatology 50: 1547–1557. 19739265 10.1002/hep.23180

[54] Holt PM, Cheng L, Cynthia Ju (2008) Identification and characterization of infiltrating macrophages in acetaminophen-induced liver injury. J Leukoc Biol. 84: 1410–1421. 10.1189/jlb.0308173 PMC2614594 18713872

[55] Ohkawa H, Ohishi N, Yagi K (1979) Assay for lipid peroxides in animal tissues by thiobarbituric acid reaction. Anal Biochem. 95: 351–358. 10.1016/0003-2697(79)90738-3 36810

