# Supplementary figures and images for: Identification of Early Biomarkers during Acetaminophen-Induced Hepatotoxicity by Fourier Transform Infrared Microspectroscopy
Source: PLoS One. 2012 Sep 19;7(9):e45521. doi: 10.1371/journal.pone.0045521 (PMC3446881; doi:10.1371/journal.pone.0045521)

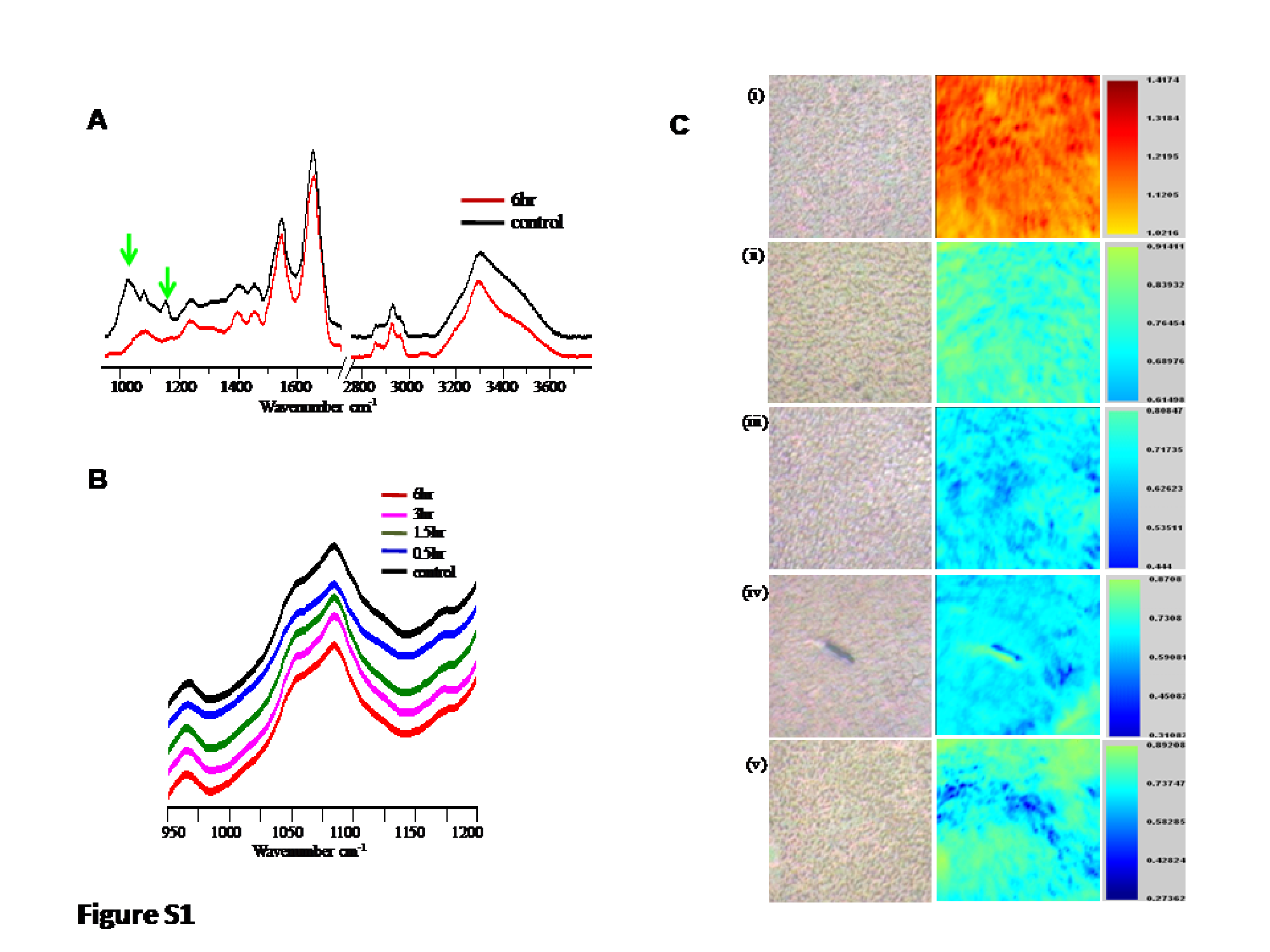

Supplement: Figure S1 — FTIR spectra (950 cm−1 to 3800 cm−1) of control and APAP treated mice livers. Arrows indicate regions of observable difference (A). Kinetic FTIR spectra (950 cm− 1 to 1200 cm− 1) of control and APAP treated mice spleen at indicated time points (B). FTIR images of control and APAP treated mice livers; both white light and glycogen distribution are shown in following order: (i) Control, (ii) 0.5 h, (iii) 1.5 h, (iv) 3 h, and (v) 6 h – post APAP treatment (C). Each image is a representative across experiments with n = 3 to 6 mice. (TIF) [file pone.0045521.s001.tif]

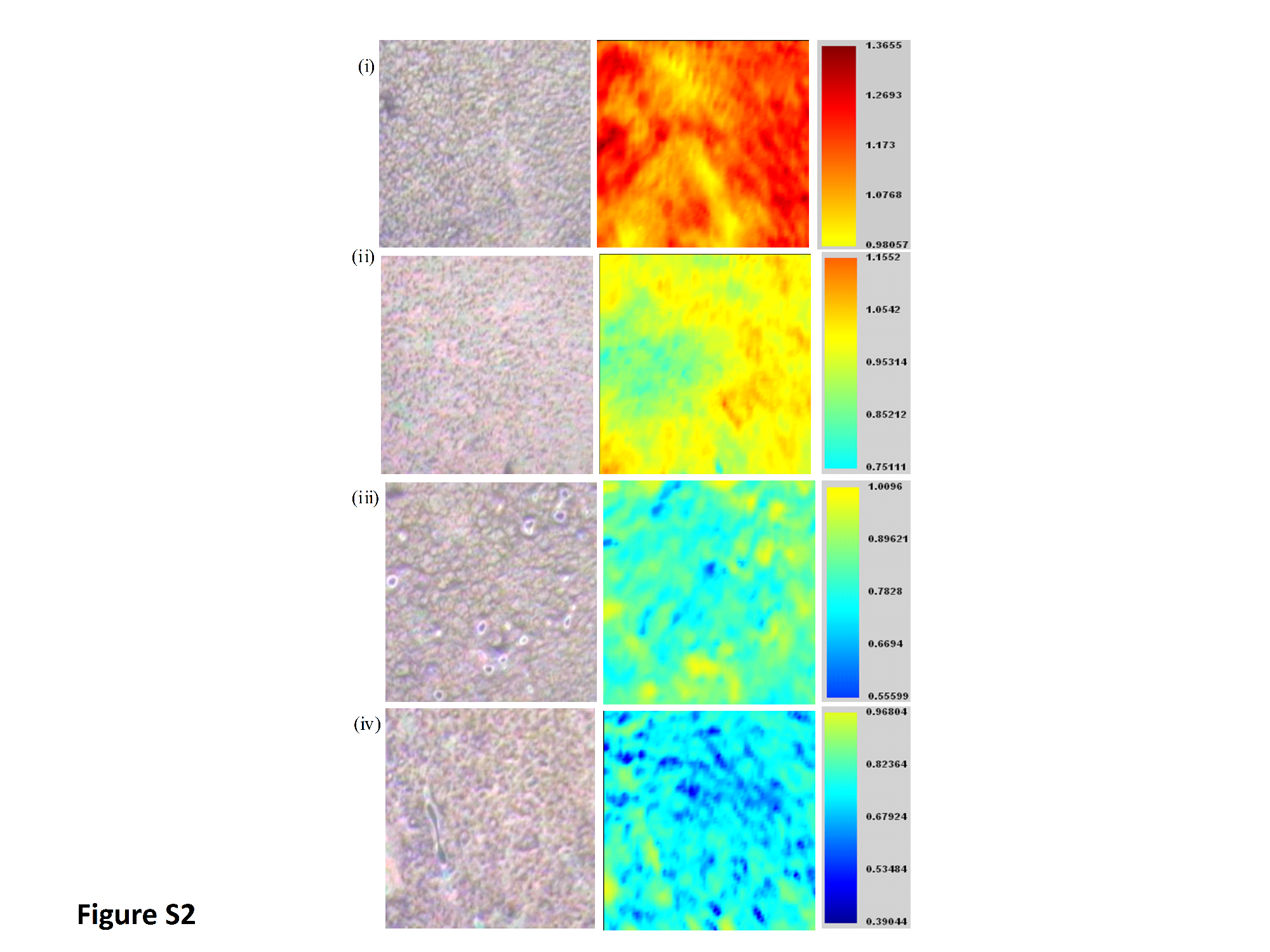

Supplement: Figure S2 — FTIR images of ∼4 µm liver sections of the following order: (i) Control, (ii) pre L-methionine treatment (−0.5 h), (iii) post L-methionine treatment (+0.5 h), (iv) APAP alone. All images represent data across many experiments with n = 5 or more mice. (TIF) [file pone.0045521.s002.tif]

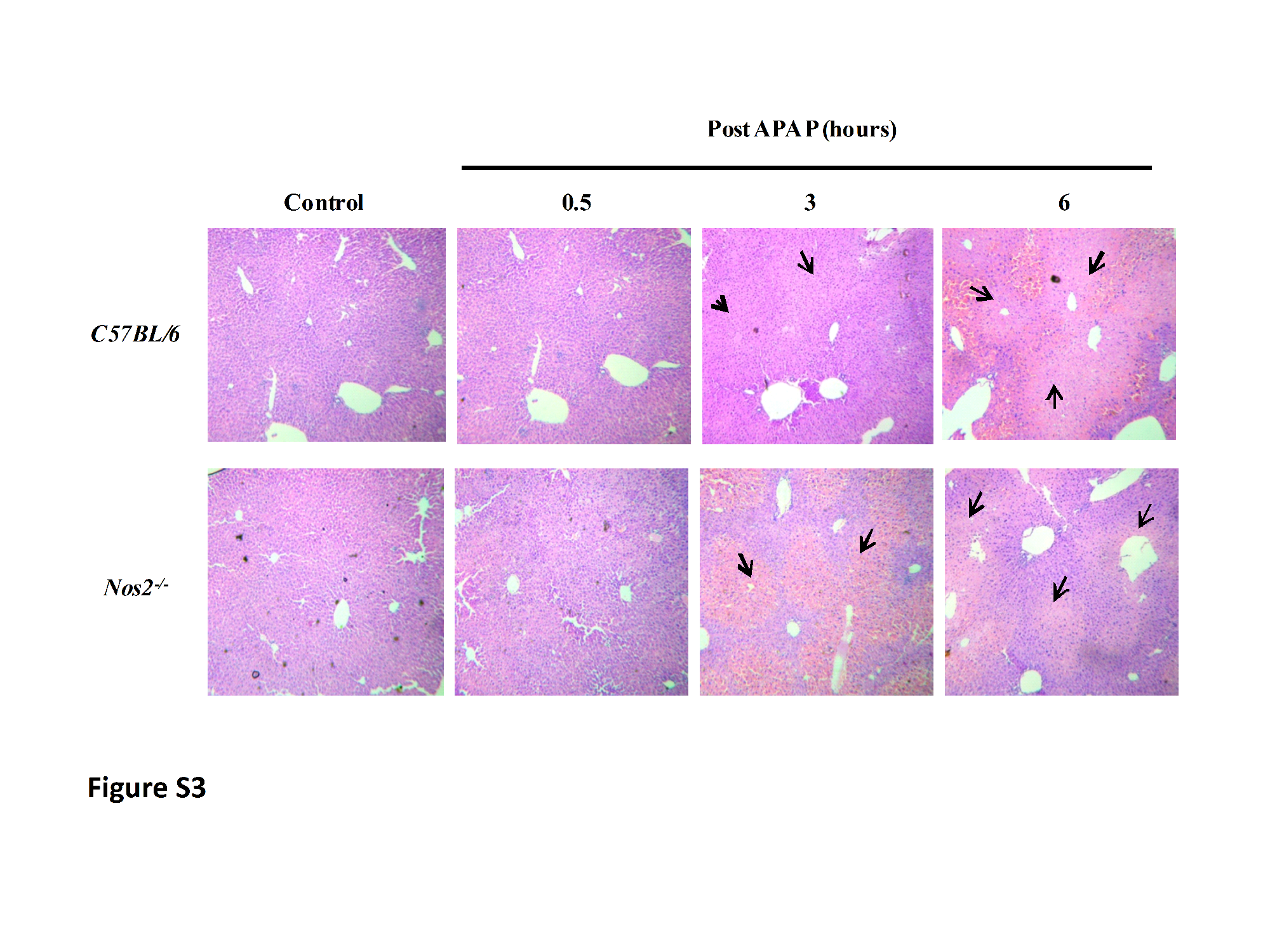

Supplement: Figure S3 — Hematoxylin and eosin stained liver sections from APAP treated and controls of C57BL/6 and Nos2−/− mice. Arrows indicate necrotic lesions. All images represent data across many experiments with n = 3 or more mice. (TIF) [file pone.0045521.s003.tif]

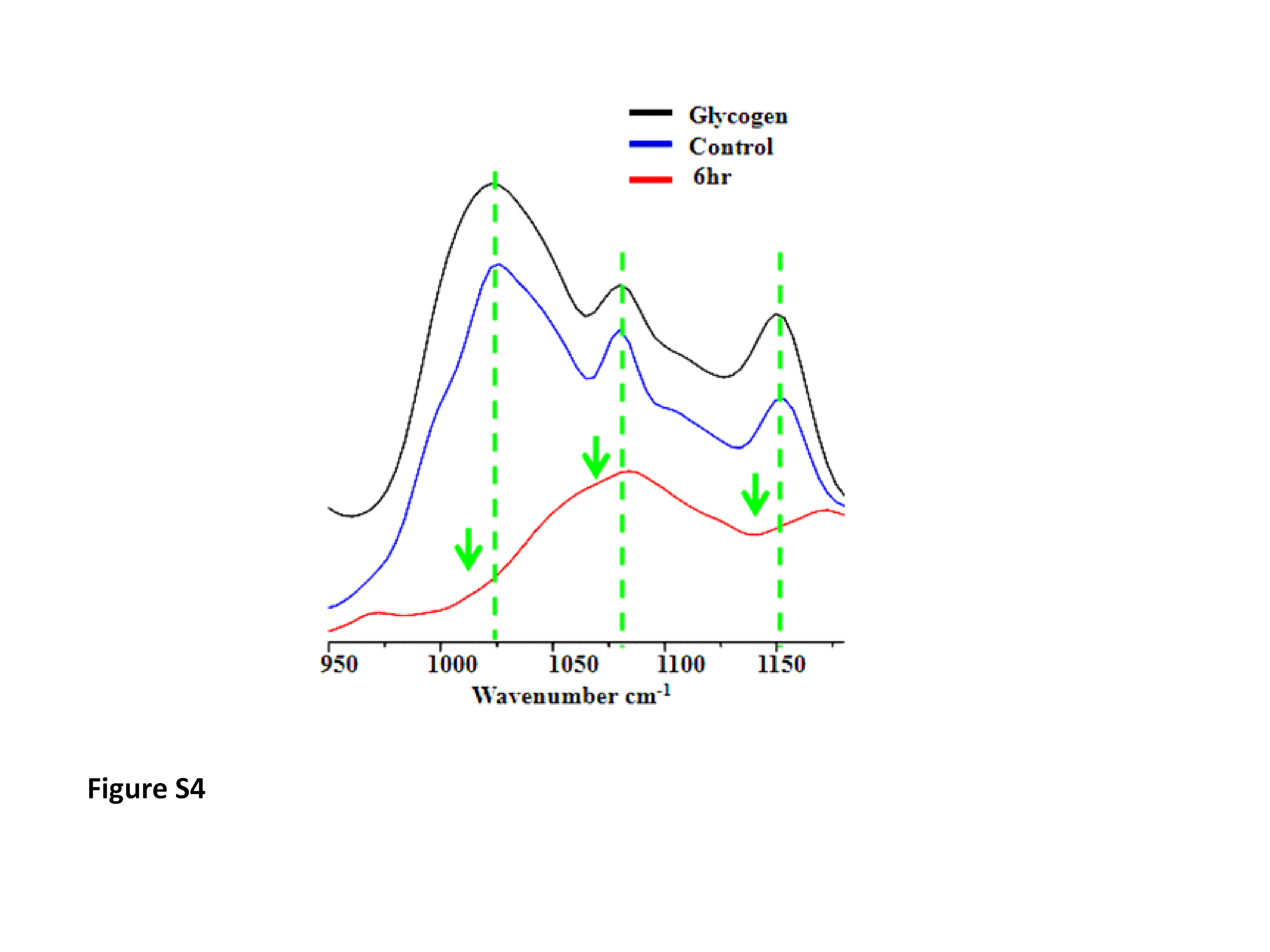

Supplement: Figure S4 — FTIR spectra (950 cm−1 to 1175 cm−1) of glycogen solution in milliQ water (black), control mice liver (blue) and 6 h post APAP treatment mice liver (red). Arrows indicate regions of observable difference. (TIF) [file pone.0045521.s004.tif]
